# Short- and long-term outcomes of laparoscopic low anterior resection with “dog ear” invagination anastomosis for mid and distal rectal cancer a propensity score matched analysis

**DOI:** 10.3389/fsurg.2022.1038873

**Published:** 2023-01-06

**Authors:** L. Zhang, Z. Xie, L. Gong, X. Lv

**Affiliations:** Department of Gastroenterology, Xuzhou Central Hospital, Xuzhou Clinical School of Xuzhou Medical College, Jiangsu, China

**Keywords:** laparoscopic low anterior resection, propensity score matching, double stapled technique, "dog-ear" invagination anastomosis, rectal cancer (RC)

## Abstract

**Background:**

The lateral intersecting margin (dog-ear) was a weak spot of the double stapled technique (DST), We designed “dog-ear” invagination anastomosis (DAIA), which could eliminate the “dog-ear” in laparoscopic anterior resection.

**Patients and methods:**

A total of 202 patients underwent elective curative LLAR + DST (*n* = 143) or LLAR + DAIA (*n* = 59) were enrolled in the study. Propensity score matching (PSM) was used to minimize the adverse effects. The clinical data between LLAR + DST and LLAR + DAIA was compared, and the effect of factors on overall survival (OS) and disease-free survival (DFS) was analyzed.

**Results:**

After PSM, 53 pairs of the LLRA + DST and LLRA + DAIA patients were enrolled in the study. The LLRA + DAIA group has a higher level (3.50 ± 1.03 vs. 2.87 ± 1.10, *P* = 0.01) of the anastomosis than that of the LLRA + DST group. Patients in LLAR + DAIA group have a lower incidence of protecting loop ileostomy compared to LLAR + DST group (20.75% vs. 5.66%, *P* < 0.05). The LLRA + DAIA patients presented better rates of LARS compare to LLRA + DST patients at 6 months (major LARS 37.74% (*n* = 20) vs. 67.93% (*n* = 36); *P* = 0.007) and 12 months (major LARS 13.21% (*n* = 7) vs. 20.37% (*n* = 11); *P* = 0.03) after surgery. The OS and DFS rates were similar (*P* > 0.05).

**Conclusion:**

Laparoscopic low anterior resection with “dog-ear” invagination anastomosis technique are well-established procedures for patients with low rectal cancer. “Dog-ear” invagination anastomosis technique may reduce the incidence of protecting loop ileostomy and significantly affect LARS score, and demonstrate a positive impact on the quality of life after surgery.

## Introduction

The lateral intersections of double-stapled anastomoses (“dog ear”) are a structural weak spot, which may increase the risk of anastomosis leakage (1). “Dog-ear” invagination anastomosis (2) is a modified anastomosis that eliminates the lateral intersections of double-stapled anastomoses and allows for a true end-to-end anastomosis. Moreover, we translate the use of CAD (The CAD is a transparent device that dilatates the anus, at the same time protects the first 2 cm of the anal canal and allows proper visualization of the anastomosis) to rectal surgery allowing better transanal introduction of the circular stapler, direct visualization of the anastomosis, easy performance of leak tests and eventually direct repair of small anastomotic defects. In theory, this technique might reduce the risk of anastomotic fistula. However, no relevant data has been reported on this aspect. The primary endpoint of this study is to reduce anastomotic leak rate after medium and low rectal cancer surgery and needing of diverting ileostomy.

Low resection syndrome is a common bowel dysfunction after anterior rectal resection and impairs quality of life. Studies have shown that 60%–90% of patients suffer from LARS (3, 4). The level of anastomosis, type of reconstruction, and anorectal compliance are known factors that influence postoperative anorectal function. Our modified anastomosis technique avoids “dog ear” residuals and reduces postoperative rectal irritation due to ischaemic areas of the anastomosis formed by the lateral intersections of double-stapled anastomoses. Theoretically, this technique may reduce the clinical symptoms of postoperative low resection syndrome.

In the present study, we designed a single-center and propensity score-matched analysis to investigate the short-term and long-term outcomes of patients with rectal cancers by comparing “dog ear” invagination anastomosis (LLAR + DAIA) to double-stapled technique (LLAR + DST).

## Material and methods

### Patients

We retrospectively analyzed a total of 202 patients with low rectal cancer who underwent Laparoscopic low anterior resection (LLAR) with curative intent at the affiliated Xuzhou Hospital of Medical College of Southeast University from January 2015 and May 2017. Fifty-nine patients received colorectal anastomosis reconstruction with “dog ear” invagination anastomosis (DAIA), the other 143 patients reconstructed with double stapler technique (DST) combined with a circular stapler. The same board-certified colorectal surgeons treated all patients, and all enrolled patients underwent radical surgery. Besides, written informed consent was obtained from each patient included in the study.

Inclusion criteria: (1) patients >18 years old; (2) undergoing laparotomic or laparoscopic rectal surgery with an anastomosis being performed; (3) in elective setting; (4) for rectal cancer (from T1N+ to T4) > 4 cm from the anocutaneous margin; (5) locally advanced rectal cancers (N+ and/or T4) undergoing neoadjuvant chemo-radiation therapy (CRT) for 5 weeks and surgery at least 8 weeks after the end of CRT; 6) without metastatic disease (M0). Exclusion criteria: (1) patients <18 years old; (2) pregnancy status; (3) rectal cancer <4 cm from the anocutaneous margin; (4) patients undergoing abdomino-perineal resection (Miles operation) or other kinds of rectal surgery without an anastomosis being performed; (5) metastatic disease (M+); (6) operations performed in emergency setting; (7) inability to cooperate with medical staff (ex because of major psychiatric diseases). The study protocol was approved by the Medical Ethics Committee of Xuzhou Central Hospital.

### Surgical technique

#### Laparoscopic low anterior resection with “dog-ear” invagination anastomosis

**Figure 1:**
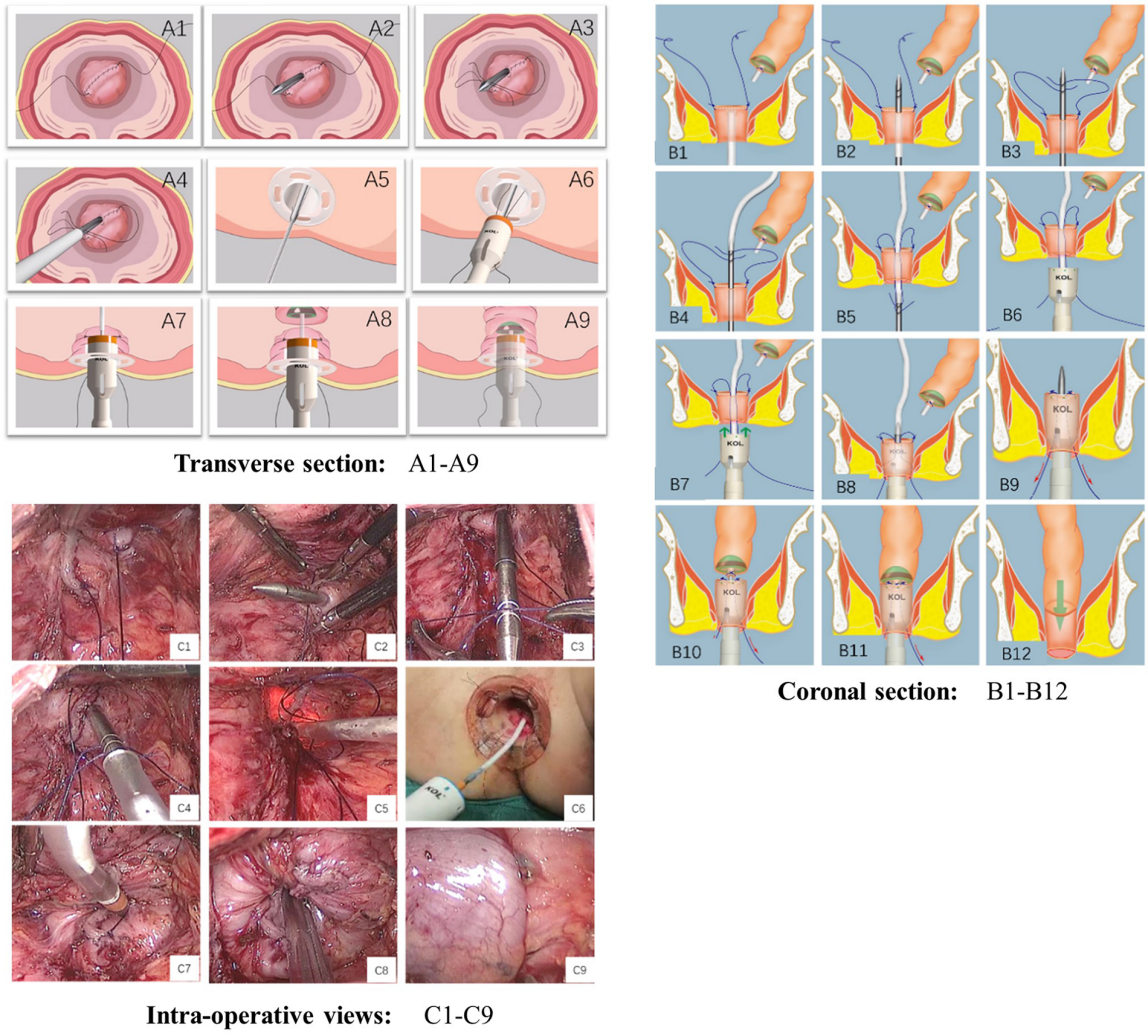
Technique of dog-ear invagination anastomosis (transverse section: **A1–A9**, coronal section: **B1–B12**, intra-operative views: **C1–C9**).

**Figure 2:**
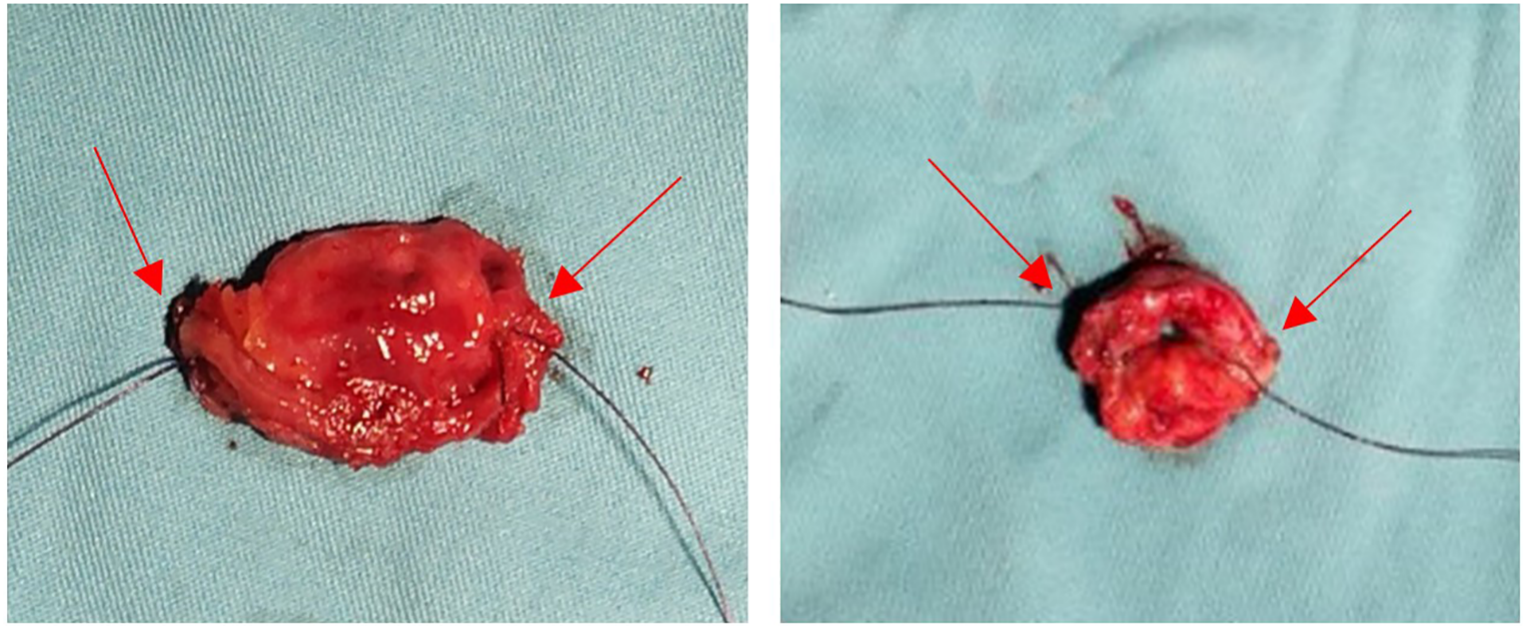
All of the dog-ears were cut down with a circular stapler (the red arrow marks the dog-ears).

After a total mesorectal resection or tumor-specific mesorectal resection of the lower rectum, the rectum is cut. A circular anal dilator (CAD) is placed in the anus. The CAD is fixed with 0-silk sutures at four base points (Transverse section: Figure 1A5) to protect the anal canal within 2 cm from the anal verge and allow 360° exploration of the inner rectal wall within 10 cm from the anal verge. The two stapled corners (“dog ear”) of the rectal stump are sutured laparoscopically with 3–0 absorbable sutures (Transverse section: Figure 1A1, Coronal section: Figure 1B1, Intra-operative views: Figure 1C1); a special puncture device (Figure 2) is inserted transanally and passed through the center of the rectal stump (Transverse section: Figure 1A2, Coronal section: Figure 1B2, Intra-operative views: Figure 1C2), with the two 3–0 absorbable sutures placed in a pre-set notches in the puncture device (Transverse section: Figure 1A3, Coronal section: Figure 1B3, Intra-operative views: Figure 1C3); a surgical drainage tube (Fr18 size medical silicone tube) is placed and attached to the tip of the puncture device (Transverse section: Figure 1A4, Coronal section: Figure 1B4, Intra-operative views: Figure 1C4); Withdraw the special puncture device and lead the surgical drainage tuber out of the anus. The two 3–0 absorbable sutures were pulled out of the anus (Transverse section: Figure 1A5, Coronal section: Figure 1B5, Intra-operative views: Figure 1C5). The circular stapler (29- or 33-mm KOL stapler, Touchstone International) was guided and inserted using the surgical drainage tube. The two tails of absorbable sutures were introduced through the traction holes of the stapler (KOL stapler, one in the left and one in the right side of the instrument) (Transverse section: Figure 1A6, Coronal section: Figure 1B6,Figure B7, Intra-operative views: Figure 1C6). The surgical drainage tube was removed laparoscopically (Transverse section: Figure 1A7, Coronal section: Figure 1B8,Figure B9, Intra-operative views: Figure 1C7). The anvil in the abdomen was connected to the KOL stapler, the sutures are tugged gradually and evenly so that the stapled corners (“dog ear”) and the anastomotic staple line are sunk into the annular anastomotic staple compartment, and the KOL stapler is tightened and fired (Transverse section: Figure 1A8,Figure A9, Coronal section: Figure Figure 1B10–Figure B12, Intra-operative views: Figure 1C8, Figure C9). The two rings were extracted from the stapler and checked for completeness. The end-to-end anastomosis can be carefully inspected under direct anoscopy. If the seal is confirmed to be intact, a defunctioning stoma was not required, and a small leak can be repaired *via* an anal suture if necessary. Figure 2 shows the complete removal of both dog-ears with a circular stapler.

#### Laparoscopic low anterior resection with double staple technique

laparoscopic low anterior rectal resection is performed according to the TME principles. The rectum is transected with a linear cutting stapler (Johnson EC60A) at the lower margin of the intended resection. A circular stapler anvil was then inserted through a small colotomy on the contralateral side of the mesocolon and secured by a purse-string suture. A 28 mm or 30 mm intraluminal stapler (Johnson CCS) is used for the anastomosis.

### Observation indicators

1. The primary outcome of this study:

#### Anastomotic leak incidence

We adopted the description of AL recently published by Rahbari NN and van Helsdingen CP (5, 6), as one of the following: postoperative peritonitis found at re-operation, fecaloid drain, fecal material from the wound, extravasation of contrast on enema, the presence of air or fluid in the anastomotic region or pelvic abscesses visualized by CT scan.

2. The second outcome of this study:
  (1) **Diverting ileostomy (DI)**

Each patient suffering from a primary leak after air insufflation underwent anastomotic repair and diverting ileostomy.

(2) **Anorectal function after laparoscopic low anterior resection**

The anorectal function was evaluated using the LARS scores at 6 and 12 months after surgery

3. Other outcome of this study our
  (1) **Surgical outcomes** included the duration of operation, intraoperative blood loss, Distal resection margin (DRM, cm), distance from the anastomosis to the dentate line (cm), the height of doughnuts.
  (2) **Postoperative complications:** The total number of complications occurred within 30 days after surgery or during the patient's hospital stay. Postoperative complications are counted according to the internationally accepted Clavien-Dindo surgical complication grading system.
  (3) **Postoperative survival:** the survival rate to the endpoint of follow-up after surgery.

### Method of follow-up

Patients are followed up from the end of treatment until September 2020 by outpatient visits. The duration of follow-up ranged from 0.9 to 6.4 years, with a median duration of 3.7 years.

### Propensity score matching analysis

Propensity score matching analysis was performed to minimize the selection bias originating from different patient and tumor characteristics. A propensity score was calculated using a logistic regression model with the operative procedure (LLAR + DAIA group or LLAR + DST group) as an objective variable; age, gender, BMI, preoperative HGB level, preoperative albumin level, ASA class, comorbidity, previous abdominal surgery, tumor location, and cTNM stage as explanatory variables. The LLAR + DAIA and LLAR + DST groups were matched according to the propensity scores using the nearest neighbor matching in a 1:1 ratio without replacement, and a caliper width of 0.2 SD was specified. After PSM, 53 patients in the LLAR + DAIA and LLAR + DST groups were included in the final analyses.

### Statistical analysis

All data were statistically processed with R.4.1.0 software. The measurement data were expressed according to the data type, with mean ± standard deviation if normally distributed or chi-squared, and median and interquartile spacing when not normally distributed or chi-squared. The t-test was used for measurement data, the *χ*^2^ test was used to compare count data, and the rank-sum test was used for rank data. Propensity score matching (PSM) was carried out by logistic regression to reduce the effects of selection bias in these two groups. The matching ratio was 1 : 1, and the covariates included age, gender, BMI, preoperative HGB level, preoperative albumin level, ASA class, comorbidity, previous abdominal surgery, tumor location, and cTNM stage. Survival curves were plotted using the Kaplan-Meier method, and differences were analyzed using the log-rank test (Log-Rank). Survival analyses were first performed using one-way analysis of variance after which single factors with *P* < 0.2 or substantiated by evidence were further included in Cox regression for multi-factor analysis. The test level was set as a two-sided test, and differences were considered statistically significant at *P* < 0.05.

## Results

Between January 2015 and May 2017, a total of 202 patients who underwent elective curative LLAR + DST (*n* = 143) or LLAR + DAIA (*n* = 59) were enrolled in the study. Baseline characteristics of the entire study sample are outlined in Table 1. There were statistically significant imbalances between the LLAR + DST and LLAR + DAIA groups for ASA, comorbidity, Preoperative HGB, and Preoperative albumin.

**Table 1:** Clinical and pathological characteristics of elderly patients with colorectal cancer before and after matching.

Varibles	Total cohort			Matched cohort		
	LLAR + DST (*n* = 143)	LLAR + DAIA (*n* = 59)	*P*	LLAR + DST (*n* = 53)	LLAR + DAIA (*n* = 53)	*P*
Age (years)*	60.26 ± 11.12	61.88 ± 10.99	0.31	60.68 ± 10.91	61.84 ± 10.89	0.22
Gender			0.97			0.53
Male	99 (69.2%)	40 (67.8%)		38 (71.7%)	34 (64.15%)	
Female	44 (30.8%)	19 (32.2%)		15 (28.3%)	19 (35.85%)	
Preoperative HGB (g/l)*	117.41 ± 24.50	125.69 ± 25.16	0.035	120.15 ± 21.76	122.04 ± 22.88	0.81
Preoperative albumin (g/l)*	33.06 ± 4.35	36.12 ± 3.24	0.03	35.27 ± 3.92	36.21 ± 3.57	0.68
Body mass index (kg/m2)*	23.54 ± 3.65	23.35 ± 3.54	0.74	23.58 ± 3.68	23.31 ± 3.54	0.69
ASA score			0.04			0.55
I–II	106 (74.1%)	52 (88.1%)		45 (84.91%)	48 (90.57%)	
III–IV	37 (25.9%)	7 (11.9%)		8 (15.09%)	5 (9.43%)	
Comorbidity			0.04			1
Yes	58 (37.9%)	29 (55.8%)		25 (47.17%)	25 (47.17%)	
No	95 (62.1%)	23 (44.2%)		28 (52.83%)	28 (52.83%)	
cTNM stage*			0.34			0.34
I–II	90 (62.9%)	42 (55.8%)		44 (83.02%)	39 (73.58%)	
III	53 (37.1%)	17 (44.2%)		9 (16.98%)	14 (26.42%)	
Tumor distance from anal verge (cm)	8.92 ± 2.86	8.82 ± 3.04	0.88	8.79 ± 2.42	8.64 ± 2.75	0.81
Previous abdominal surgery			0.58			0.82
Yes	36 (25.2%)	12 (20.3%)		11 (20.75%)	13 (24.53%)	
No	107 (74.8%)	47 (79.7%)		42 (79.25%)	40 (75.47%)	
Preoperative CEA (ng/ml)			0.91			0.44
<−5	119 (83.2%)	48 (81.4%)		42 (79.25%)	46 (86.79%)	
>5	24 (16.7%)	11 (18.6%)		11 (20.75%)	7 (13.21%)	

* Values given as mean ± SD; HGB, hemoglobin; ASA, American society of anesthesiologists; cTNM, clinic tumor nodes metastasis; CEA, carcinoembryonic antigen.

** Evaluated based on 7th edition of AJCC.

### Study population after propensity score matching

Details of the 1 : 1 PSM process are described in the Methods section. 53 marched pairs were selected through propensity scoring. There were no significant group differences in the baseline demographic, clinical, or tumor variables (see Supplementary Figures S1, S2, Table S1).

### Operative and postoperative outcomes

Operative and postoperative outcomes are displayed in Table 2. No differences were observed in terms of hospital stay (9.21 ± 3.04 vs. 8.92 ± 2.84, *P* = 0.49), and estimated blood loss (68.25 ± 24.88 vs. 75.75 ± 24.06, *P* = 0.68) between the LLAR + DST and LLAR + DAIA approaches. There was a significant difference in operative time between the two groups. The LLAR + DAIA group demonstrated a longer operative time (148.75 ± 10.31 vs. 117.52 ± 11.92, *P* = 0.008) than the LLAR + DST group. But this time can be minimized with the development of the learning curve. Patients in LLAR + DAIA group have a lower incidence of protecting loop ileostomy compared to LLAR + DST group (20.75% vs. 5.66%).

**Table 2:** Perioperative outcomes and pathologic outcomes in patients with rectal cancer operated on by LLAR + DST or LLAR + DEIA after propensity score matching (*n* = 106).

Variables	LLAR + DST (*n* = 53)	LLAR + DAIA (*n* = 53)	*p*
Surgical outcome			
Operative time (min)*	117.52 ± 11.92	148.75 ± 10.31	**0.008**
Intraoperative blood loss (ml)*	68.25 ± 24.88	75.75 ± 24.06	0.68
Protecting loop ileostomy, *n* (%)			**0.04**
Yes	11 (20.75%)	3 (5.66%)	
No	42 (79.25%)	50 (94.34%)	
Postoperative complications			
Incisional infection	6 (11.32%)	4 (7.55%)	0.74
Anastomotic leak	5 (9.43%)	2 (3.77%)	0.68
Ileus	5 (9.43%)	8 (15.09%)	0.56
Blood transfusion postoperatively	3 (5.66%)	2 (3.77%)	1
Intra-abdominal hemorrhage	1 (1.88%)	2 (3.77%)	1
Pelvic abscess	2 (3.77%)	3 (5.66%)	1
Intestinal–vaginal fistula	0 (0)	1 (1.88%)	1
Postoperative complication (ClavienDindo classification), *n* (%)			
I–II	14 (26.4%)	15 (28.3%)	1
III–V	6 (11.3%)	4 (7.55%)	0.74
Mortality during hospitalization, *n* (%)	0	0	1
Mean postoperative LOS (day)*	9.21 ± 3.04	8.92 ± 2.84	0.49
Cost ($)*	9491.75 ± 368.55	9593.03 ± 251.37	0.62
Pathological outcome			
Harvested lymph node*	18.71 ± 10.05	17.93 ± 9.84	0.49
Positive DRM, *n* (%)	2 (3.77%)	1 (1.89%)	1
Distal resection margin (DRM) (cm)*	2.28 ± 1.32	2.78 ± 0.81	0.54
Distance from the anastomosis to the dentate line (cm)*	3.50 ± 1.03	2.87 ± 1.10	**0.01**
The height of doughnuts	0.83 ± 0.19	1.05 ± 0.36	**0.02**
LARS score classification (6 months after surgery), *n* (%)			**0.007**
No LARS	6 (11.32%)	9 (16.98%)	
Minor LARS	11 (20.75%)	24 (45.28%)	
Major LARS	36 (67.93%)	20 (37.74%)	
LARS score classification (12 months after surgery), *n* (%)			**0.03**
No LARS	15 (27.78%)	28 (52.83%)	
Minor LARS	28 (51.85%)	18 (33.96%)	
Major LARS	11 (20.37%)	7 (13.21%)	
Conversion to open surgery, *n* (%)	0	0	1

* Values given as mean ± SD.

Overall morbidity was similar between the groups, and Anastomotic leakage occurred in a total of six patients (11.32%). The four and two anastomotic leakages occurred in the LLAR + DST group and LLAR + DAIA group, respectively. The anastomotic leakage rate in the LLAR + DAIA group was lower than that in the LLAR + DST group (3.77% vs. 9.43%, *P* = 0.68). Patients in LLAR + DAIA group have a lower incidence of protecting loop ileostomy compared to LLAR + DST group (20.75% vs. 5.66%, *P* < 0.05). In this study, the level of anastomosis was measured in a sagittal plane of postoperative CT images. The level of anastomosis was higher in the LLAR + DAIA group than in the LLAR + DST group (3.50 ± 1.03 vs. 2.87 ± 1.10, *P* = 0.01), and the height of doughnuts was larger than in the conventional anastomosis group (1.05 ± 0.36 vs. 0.83 ± 0.19, *P* = 0.02).

### Pathologic outcomes

No group differences were observed for the pathological outcome in the positive DRM (3.77% vs. 1.89%, *P* = 1) and harvested lymph node (18.71 ± 10.05 vs. 17.93 ± 9.84, *P* = 0.49) between the LLAR + DST group and LLAR + DAIA group.

### Endoscopic evaluation of anastomosis at six-months follow-up

**Figure 3:**
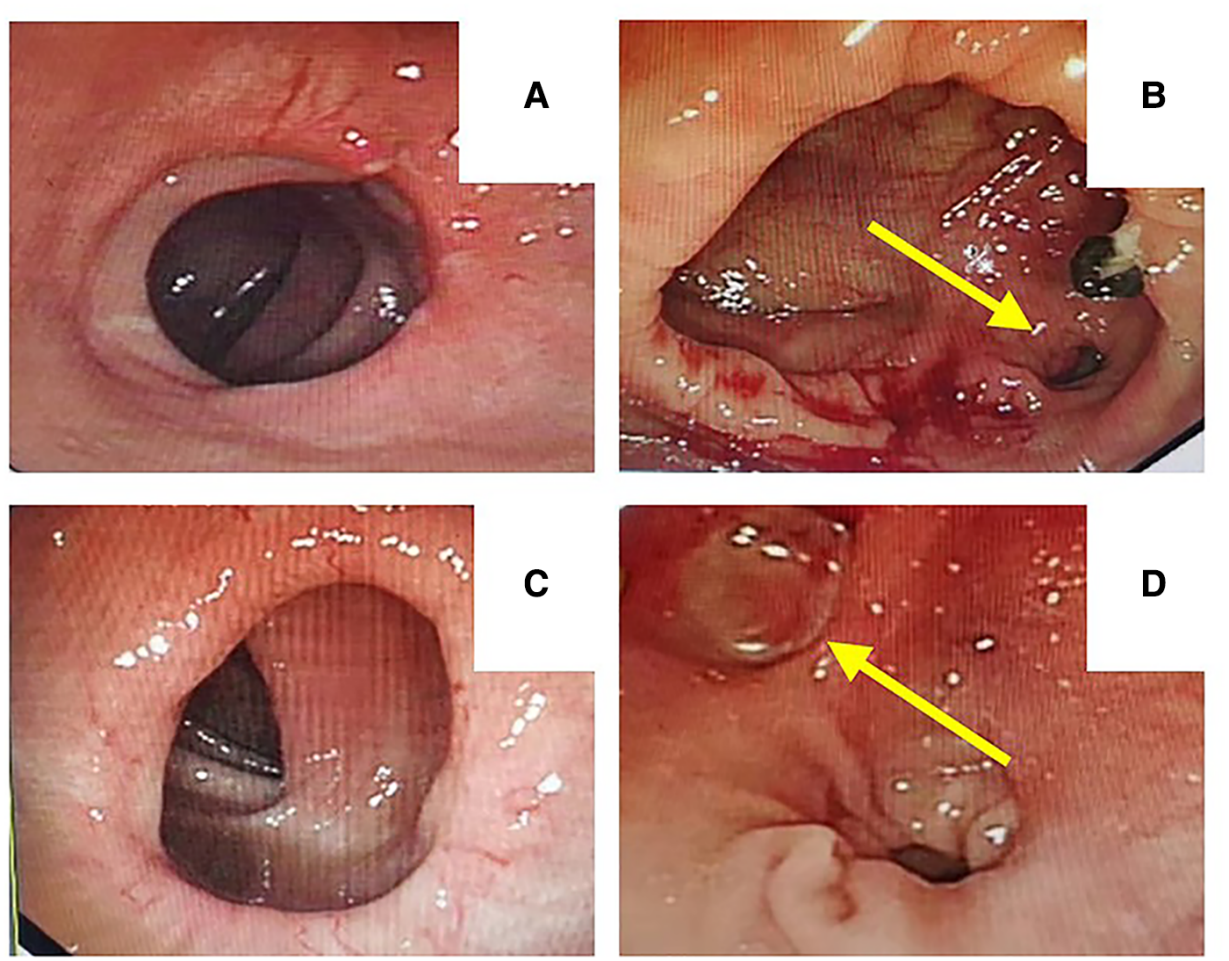
Endoscopic evaluation of anastomosis at 6-month follow-up [**A, C**: “dog-ear” invagination anastomosis had no “dog-ears” left, **B, D**: double-staple technique with 1 or 2 dog-ears left (yellow arrow)].

Six months after surgery, all patients underwent endoscopic examination. Patients who underwent the double-staple anastomosis with 1 or 2 dog-ears left (Figures 3B,D), and patients with “dog-ear” invagination anastomosis had no “dog-ears” left (Figures 3A,C).

### LARS score

We evaluated the LARS scores at six months and twelve months after closure of the ileostomy or after resection of the rectum (in cases where no stoma was created). LARS symptoms were present in 44 (83.02%) patients in the LLAR + DAIA group and 47(88.68%) in the LLAR + DST group at six months after surgery. The proportions of no LARS, minor LARS, and major LARS were significantly different between the LLAR + DST and LLAR + DAIA groups at six months(major LARS 37.74%(*n* = 20) vs. 67.93% (*n* = 36); minor LARS 45.28% (*n* = 24) vs. 20.75% (*n* = 11); *P* = 0.007) and twelve months(major LARS 13.21% (*n* = 7) vs. 20.37% (*n* = 11); minor LARS 33.96% (*n* = 18) vs. 51.85% (*n* = 28); *P* = 0.03) after surgery. It showed that median LARS scores were significantly higher in the LLAR + DST group compared with the LLAR + DAIA group at six months (33 [IQR 9] vs. 28 [IQR 10], *P* = 0.04) and twelve months (26 [IQR 9] vs.20 [IQR 14], *P* = 0.02) after surgery.

### Survival analysis

**Figure 4:**
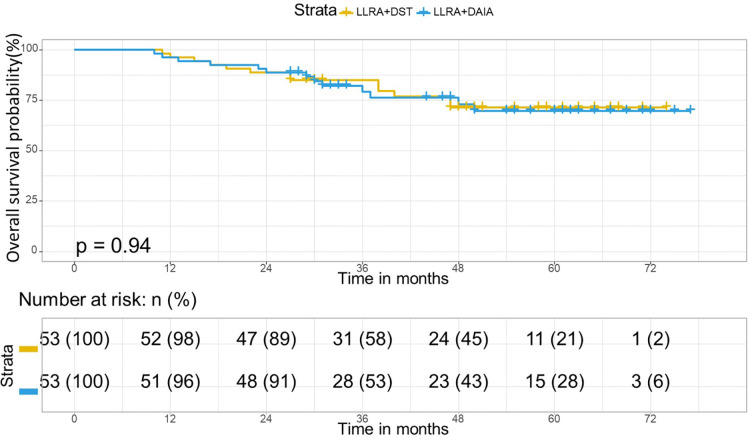
Overall survival curve in matched cohort of LLAR + DST and LLAR + DAIA groups. In the matched cohort, in the LLAR + DST group, 3- year and 5-year Overall survival rates were 79.2 and 71% respectively and they were 79 and 69.4% respectively in the LLAR + DEIA group. There was no significant difference between the LLAR + DST and LLAR + DEIA groups (*P* = 0.95).

**Figure 5:**
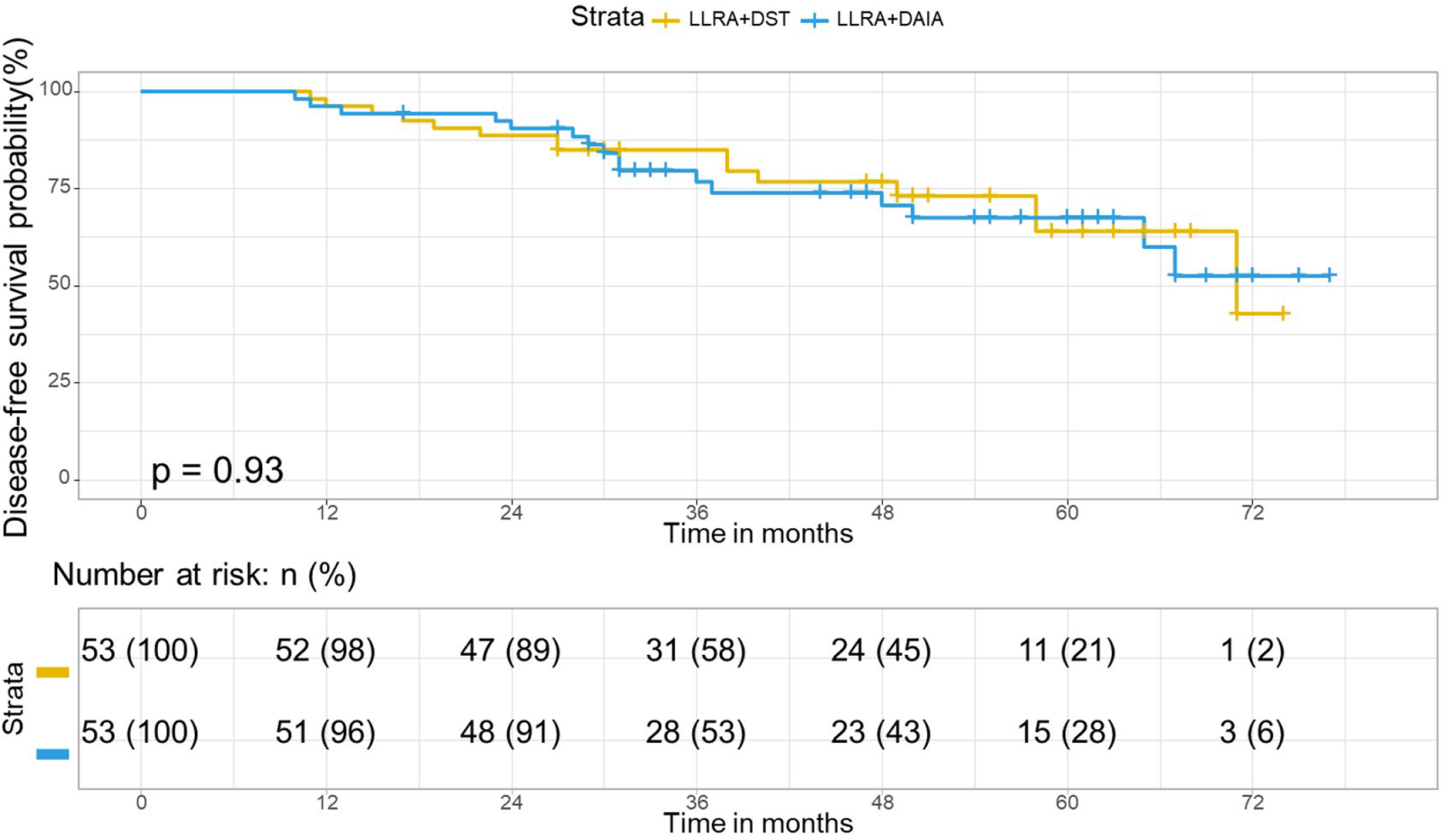
Disease-free curve in matched cohort of LLAR + DST and LLAR + DAIA groups. In the matched cohort, in the LLAR + DST group, 3- year and 5-year disease-free survival rates were 79.2 and 63.7% respectively and they were 76.5 and 67.2% respectively in the LLAR + DAIA group. There was no significant difference between the LLAR + DST and LLAR + DAIA groups (*P* = 0.94).

The median follow-up period in the matched cohort was 44 months (range, 10–77 months; LLAR + DAIA group: 36 months; LLAR + DST group: 47 months). 26 of the 106 patients died (24.5%), and 31 of the 106 patients had a local recurrence or distant metastasis (29.2%). In the matched cohort, the Kaplan curves showed no statistically significant difference in OS (*P* = 0.95) and DFS (*P *= 0.94) between the two groups. Besides, the 3- and 5-year OS rates in the LLAR + DST group were 79.2% and 71%, respectively, and those in the LLAR + DAIA group were 79% and 69.4%, respectively (Figure 4). In addition, the 3-year DFS and 5-year DFS rates were 79.2% and 63.7% respectively in the LLAR + DST group, and they were 76.5% and 67.2%, respectively (Figure 5) in the LLAR + DAIA group.

At multivariate analysis, the OS was significantly affected by CEA level (HR: 2.12; 95% CI: 1.18–4.64; *P* = 0.031), pTNM stage (HR: 3.48; 95% CI: 1.41–8.60; *P* = 0.006). Besides, DFS was significantly affected by the CEA level (HR: 1.82; 95% CI: 1.35–4.03; *P* = 0.038), pTNM stage (HR: 3.36; 95% CI: 1.24–9.12; *P* = 0.017) (Table 3).

**Table 3:** Univariate and multivariate analysis for overall survival and disease-free survival in matched cohorts.

Variable	Overall survival				Disease-free survival			
	Univariate analysis		Multivariate analysis		Univariate analysis		Multivariate analysis	
	Hazard ratio (95% CI)	*P*	Hazard ratio (95% CI)	*P*	Hazard ratio (95% CI)	*P*	Hazard ratio (95% CI)	*P*
**Age, continuous**	0.99 (0.96–1)	0.64			0.99 (0.95–1)	0.66		
**ASA**								
I–II	Reference				Reference			
III–IV	1.3 (0.38–4.3)	0.69			1.5 (0.43–4.9)	0.51		
**Sex**		0.75						
Female	Reference				Reference			
Male	1.2 (0.49–2.7)				1.2 (0.48–3.1)			
**Pre-treatment BMI, categorical**								
<18.5 kg/m^2^	Reference				Reference			
18.5–24.9 kg/m^2^	1.36 (0.64–2.42)	0.58			1.42 (0.69–2.51)	0.68		
≥25.0 kg/m^2^	1.45 (0.71–2.67)	0.53			1.53 (0.82–2.85)	0.35		
**Postoperative LOS, days**		0.89				0.69		
≤7	Reference				Reference			
>7	1.14 (0.49–2.31)				1.12 (0.43–1.93)			
**Comorbidity > 1**		0.36				0.52		
No	Reference				Reference			
Yes	1.63 (0.69–4.25)				1.41 (0.48–3.43)			
**pTNM stage**		**0.008**	** **	**0.006**	** **	**0.003**	** **	**0.017**
I–II	Reference		Reference		Reference		Reference	
III–IV	3.9 (1.8–8.5)		3.48 (1.41–8.60)		3.5 (1.5–7.9)		3.36 (1.24–9.12)	
**Harvested lymph node**		0.37				**0.031**		0.081
>12	Reference				Reference		Reference	
≤12	0.82 (0.41–1.82)				2.21 (1.13–4.52)		1.79 (0.72–3.42)	
**Operative type**		0.96				0.81		
LLAR + DST	Reference				Reference			
LLAR + DAIA	1 (0.47–2.2)				1.1 (0.49–2.5)			
**Preoperative CEA (ng/ml)**	** **	**0.006**	** **	**0.031**	** **	**<0.001**	** **	**0.038**
<5	Reference		Reference		Reference		Reference	
≥5	1.92 (0.81–3.84)		2.12 (1.18–4.64)		2.46 (1.92–4.59)		1.82 (1.35–4.03)	
**Complications (Clavien Dindo III–V)**		0.87				0.92		
No	Reference				Reference			
Yes	1.24 (0.54–3.26)				1.18 (0.37–3.02)			

pTNM Pathology tumor nodes metastasis; LLAR + DST Laparoscopic low anterior resection with double stapler technique LLAR + DAIA Laparoscopic low anterior resection with “dog-ear” invagination anastomosis.

## Discussion

**Figure 6:**
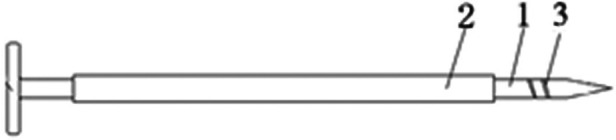
Special puncture device, with two notches (**1:** puncture needle body **2:** sleeve fitted to the outer side of the puncture needle body **3:** notches located in the body of the puncture needle).

Roumen et al. (7) demonstrated in animal studies that the lateral intersections of double-stapled anastomoses (“dog ear”) are a structural weak spot, and the bursting pressure of double-stapled anastomoses is significantly lower than that of circular-stapled anastomoses. Therefore, they suggest that a new device should be developed to easily make circular anastomosis without leaving behind any risky dog ears in the deep pelvis after a low anterior resection. In the present study, with the special puncture device (Figure 6) we designed, our modified technique, dog-ear invagination anastomosis, facilitates colorectal anastomoses at a lower level and eliminates the dog-ear in laparoscopic anterior resection of the rectum. There was no significant difference in intraoperative blood loss, pathological outcomes, postoperative recovery, and postoperative complication between the two groups. It suggests that the dog-ear invagination anastomoses at a lower level were technically feasible and safe from aspects of the short outcome.

Anastomotic leak is the most severe complication of Laparoscopic low anterior resection, and many factors (8, 9) are known that influence disruption or dehiscence. The most crucial factor is the distance of the anastomosis from the anal verge: the lower the anastomosis, the more leakages occur. Our clinical data show that the “dog-ear” invagination anastomose group has a higher level (3.50 ± 1.03 vs. 2.87 ± 1.10, *P* = 0.01) of the anastomosis than that of the double stapler technique group. That's because the KOL stapler has a large volume staple compartment, which makes the dog-ear invagination anastomose group has a higher height of doughnuts (1.05 ± 0.36 vs. 0.83 ± 0.19, *P* < 0.05) than the double stapler technique group. It can be used as an effective complement to the length of the distal surgical margin, thus eliminating the need to pursue a lower cutting, increasing the level of the colorectal anastomosis, which to some extent reduces the difficulty of cutting rectum and the damage to the sphincter caused by excessive stretching. As the “dog-ear” invagination anastomose removes not only the “dog ears”, a structural weak spot, but also retains a higher level of anastomosis. It theoretically reduces the risk of anastomotic leakage. Our clinical data also show that the incidence of anastomotic leakage is lower in the “dog-ear” invagination anastomose group than in the double anastomosis group. However, there is no statistically significant difference, perhaps due to our small sample size.

In many cases of low anterior resection, a protecting loop ileostomy is often constructed with the aim of diverting faeces. However, the role of using a loop ileostomy for faecal diversion in patients undergoing rectal resection and anastomosis is controversial. Indeed, there seems to be better evidence that the presence of a stoma will lessen the extent of sepsis and morbidity if leakage does occur (10). Complications following a stoma range from minor complications requiring only local care to devastating complications requiring reoperation and prolonged hospitalisation (11, 12). How to reduce the incidence of prophylactic stomas may be a problem for surgeons in the future. An end-angle invagination anastomosis can eliminate the end angle, while intraoperative use of CAD for anastomotic inspection and remediation provides a measure of assistance to surgeons in not selecting a stoma. In our study, patients in LLAR + DAIA group have a lower incidence of protecting loop ileostomy compared to LLAR + DST group(20.75% vs. 5.66%, *P* < 0.05).

“Low anterior resection syndrome” (LARS) has been reported to occur in up to 80% of patients after low anterior resection (LAR) with a detrimental impact on their quality of life (13, 14). When determining the total LARS score, there were no significant differences between the cases reconstructed with DEIA and DST in this study. When the LARS score was calculated and categorized into groups of “no”, “minor,” or “major LARS”, the number with major LARS after LLAR + DEIA was less than those observed after LLAR + DST at the 6 and 12 months, it suggests that the patients with LLAR + DEIA may have better anorectal function compared to LLAR + DST. Various risk factors (15, 16) have been identified in LARS development, such as age, sex, surgical technique, the level of anastomosis, neoadjuvant and adjuvant chemotherapy or/and radiotherapy (CRT), and postoperative complications. The diverticular effect of the dog's ear leads to anorectal irritability, which affects fecal control and defecation, resulting in discomfort such as a marked sense of urgency and increased frequency of defecation. Moreover, our datas show that the double stapler technique group has a lower level (2.87 ± 1.10 vs. 3.50 ± 1.03, *P* = 0.01) of the anastomosis than that of the “dog-ear” invagination anastomose group. All these factors together make the double stapler technique group has worse anorectal function than the “dog-ear” invagination anastomose group.

In terms of the oncologic aspects, this study's 5-year disease-free survival rate showed no significant difference between LLAR + DEIA and LLAR + DST surgical procedures (63.7% vs. 67.2%, *P* = 0.94). The 5-year overall survival rate was 69.4% for LLAR + DEIA and 71% for LLAR + DST (*P* = 0.95) surgical procedures. which is in line with the results of others (17, 18). The local recurrence rate in the LLAR + DEIA group was also acceptable as 24.52% and similar to the local recurrence of LLAR + DST(22.64%) surgery. We also assessed the possible factors that may interfere with the survival of CRC patients to understand which factors are truly associated with their survival. The variables were age, sex, TNM.stage, preoperative CEA level, comorbidity, Consistently with the previous studies (19, 20). Univariate and multivariate analysis showed that the type of surgical procedure did not affect prognosis for the 5-year disease-free and overall survival. This result can be translated that DEIA is equivalent to DST surgery for rectal cancer in terms of long-term oncologic outcomes.

This study has some limitations and selection biases inherent in any retrospective analysis. However, selection bias was reduced by propensity score matching through logistic regression. Multicenter large-scale prospective studies are needed to further investigate the risk of leaks and the impact on anorectal function between anastomoses with and without dog ears.

In summary, Laparoscopic low anterior resection with “dog-ear” invagination anastomosis technique are well-established procedures for patients with low rectal cancer. “Dog-ear” invagination anastomosis technique may reduce the risk of anastomotic fistula and significantly affect LARS score and demonstrate a positive impact on the quality of life after surgery.

## Data Availability

The original contributions presented in the study are included in the article/**Supplementary Material**, further inquiries can be directed to the corresponding author/s.
