# Supplementary figures and images for: Short- and long-term outcomes of laparoscopic low anterior resection with “dog ear” invagination anastomosis for mid and distal rectal cancer a propensity score matched analysis
Source: Front Surg. 2023 Jan 6;9:1038873. doi: 10.3389/fsurg.2022.1038873 (PMC9852756; doi:10.3389/fsurg.2022.1038873)

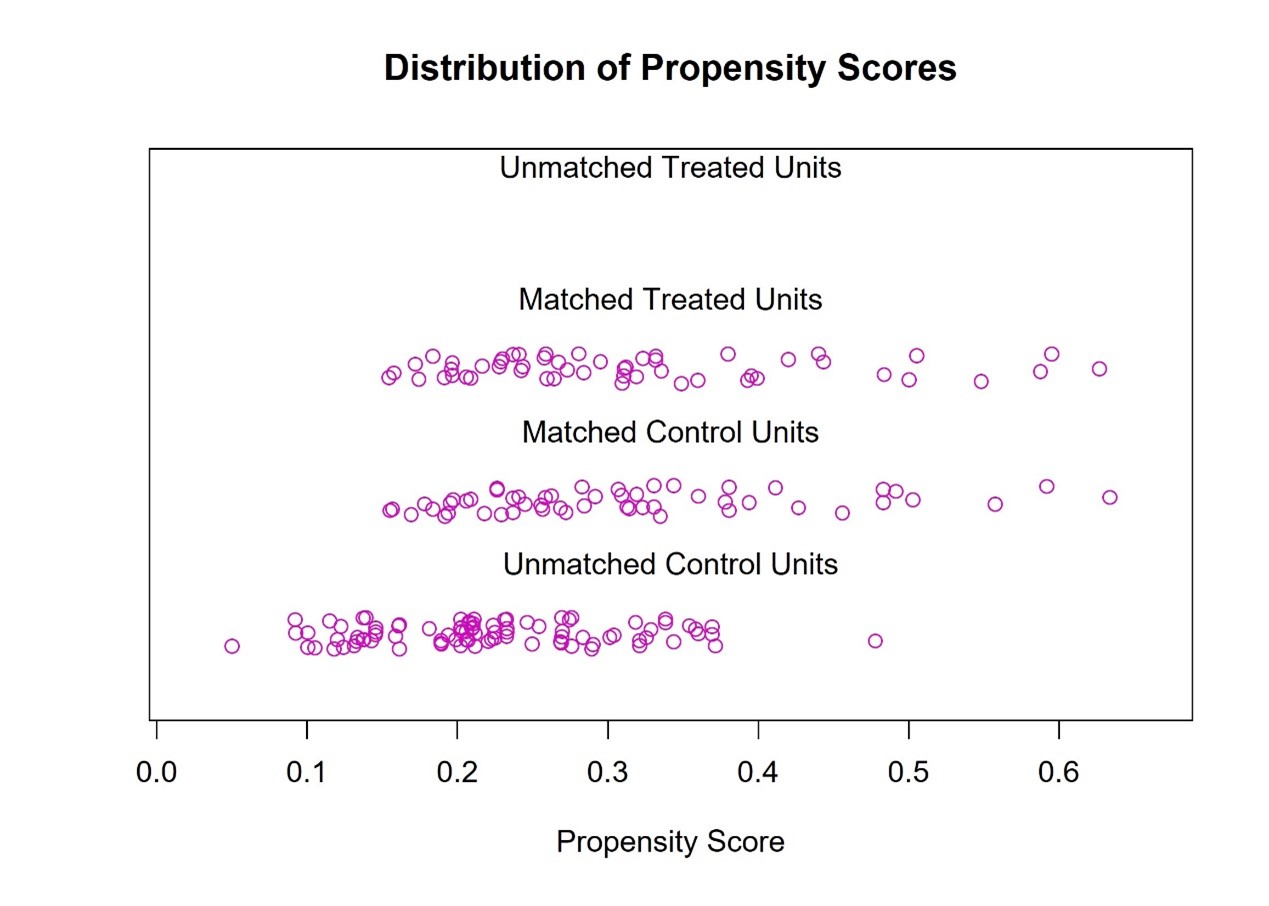

Supplement: Supplementary file 1 [file Image1.jpeg]

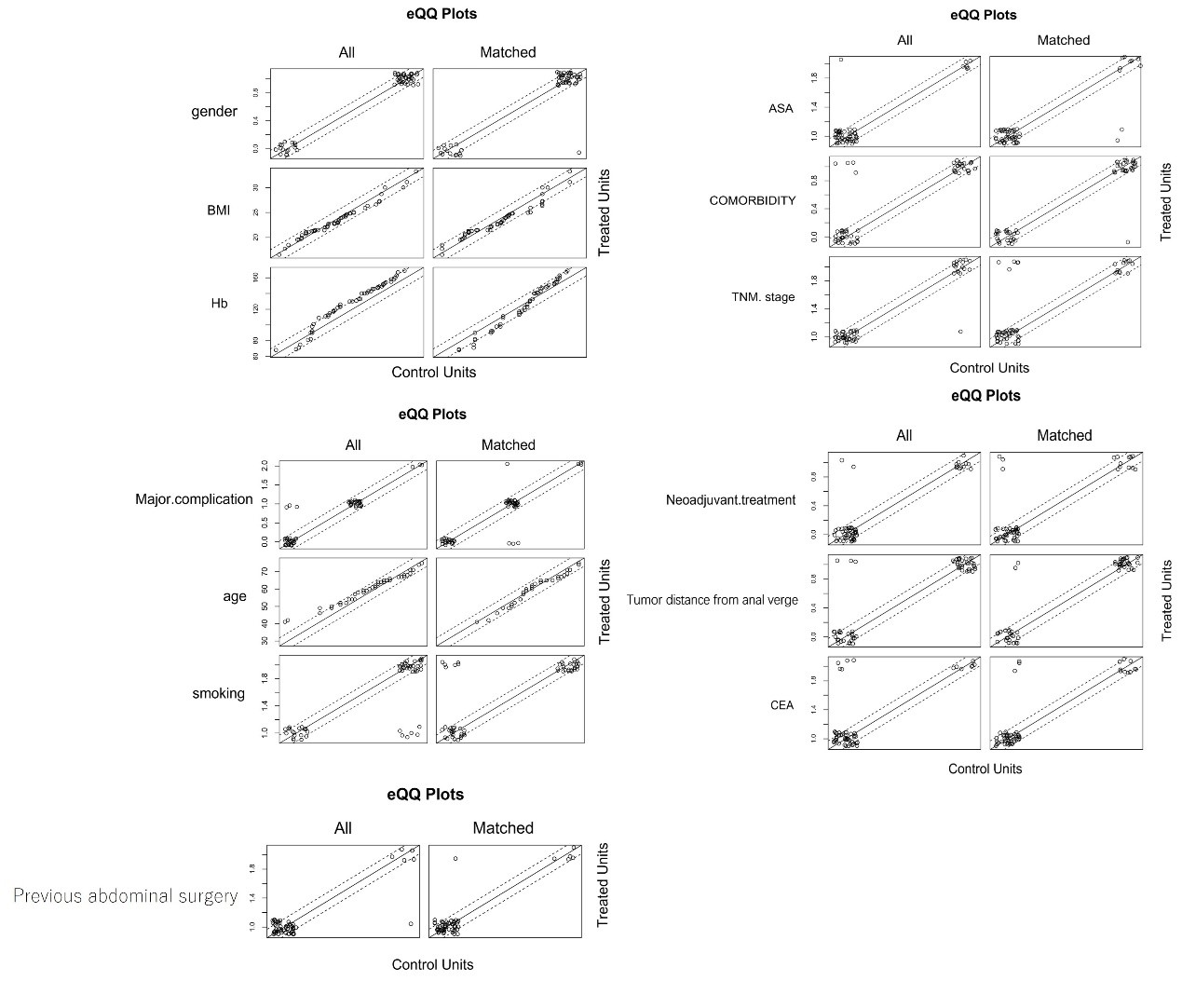

Supplement: Supplementary file 2 [file Image2.jpeg]
