# Supplementary material for: Short- and long-term outcomes of laparoscopic low anterior resection with “dog ear” invagination anastomosis for mid and distal rectal cancer a propensity score matched analysis
Source: Front Surg. 2023 Jan 6;9:1038873. doi: 10.3389/fsurg.2022.1038873 (PMC9852756; doi:10.3389/fsurg.2022.1038873)
Supplement: Supplementary file 3 [file Table1.docx]

|  | Std.Mean Diff. |
| --- | --- |
| **distance** | 0.0271 |
| Gender | 0.0822 |
| BMI | 0.0693 |
| Hb | 0.0364 |
| ASA | 0.1656 |
| Comorbidity | 0.0755 |
| TNM stage | 0.1602 |
| Major complication | 0.1259 |
| Age | 0.1183 |
| Smoking | 0.1872 |
| Neoadjuvant treatment | 0.0877 |
| Tumor distance from anal verge | 0.0383 |
| CEA | 0.0893 |
| Previous abdominal surgery | 0.0123 |

**Table S1.** Standardized mean differences between LLAR+DAIA group and LLAR+DST group after PSM
